# High-Uniformity Core-Shell Nanofibers for Semiconductor Packaging: Process Optimization and Performance Study of Airflow-Assisted Coaxial Electrospinning

**DOI:** 10.3390/mi17040463

**Published:** 2026-04-10

**Authors:** Xun Chen, Shize Huang, Rongguang Zhang, Xuanzhi Zhang, Jiecai Long, Guohuai Lin

**Affiliations:** 1 State Key Laboratory of Precision Electronic Manufacturing Technology and Equipment, Guangdong University of Technology, Guangzhou 510006, China; 2 School of Electromechnical Engineering, Guangdong University of Technology, Guangzhou 510006, China

**Keywords:** core-shell nanofiber, gas-assisted coaxial electrospinning, semiconductor application, uniformity control, response surface method

## Abstract

Semiconductor miniaturization demands stricter material uniformity. Core-shell nanofibers, promising for semiconductor packaging and flexible circuits, face application limits due to traditional coaxial electrospinning’s electric field instability—causing poor fiber diameter uniformity and challenges with high-viscosity and low-conductivity solutions. To address this, airflow-assisted coaxial electrospinning leveraged airflow-electric field synergy to enhance fiber stretching. COMSOL Multiphysics 6.4 simulated the influence of different inner diameters of the air flow nozzles on the air flow field, while the response surface method optimized parameters. At 10 kPa air pressure, 16.71 kV voltage, and a gas nozzle inner diameter of 3.42 mm, nanofibers showed regular morphology with a diameter coefficient of variation as low as 9.2%. This study enables stable preparation of highly uniform core-shell nanofibers, providing key process support for their large-scale semiconductor application and advancing flexible electronics and photodetection.

## 1. Introduction

The continuous miniaturization of semiconductor devices has placed higher requirements on material uniformity. The precise control of structural consistency directly affects the performance and reliability of devices [1,2,3,4,5,6,7]. Core-shell nanofibers can independently regulate the properties of the core and shell, such as conductivity, mechanical flexibility, and interface stability, by virtue of their unique structure, and have become potential candidate materials for semiconductor packaging and flexible circuits [8,9,10,11,12,13]. Currently, the preparation methods for core-shell structured nanofibers include template synthesis, self-assembly, and the traditional coaxial electrospinning method [14,15,16,17,18]. However, these methods are relatively inefficient, and the traditional coaxial electrospinning technology has key limitations that hinder its large-scale application: unstable electric fields lead to poor fiber diameter uniformity, and its adaptability to high-viscosity or low-conductivity precursor solutions is limited, which restricts the use of functional materials required for advanced semiconductor applications [19,20,21,22].

To address these challenges, gas-assisted coaxial electrospinning (GACES) has been proposed as a hybrid method [23,24]. It enhances fiber stretching and morphological control through the synergistic effect of electrostatic repulsion and directional airflow. Although previous studies have verified the potential of GACES, systematic research on the optimization of process parameters and their influence on the uniformity of core-shell fibers is still relatively limited.

This study combines COMSOL finite element simulation and the response surface method (RSM) to systematically optimize the GACES process parameters. The influence of the inner diameter of the gas nozzle on the coaxial needle hole on the flow field distribution is analyzed through COMSOL simulation, revealing the potential mechanism of fiber stretching and uniformity. Subsequently, the RSM is used to optimize key process parameters, including gas pressure, applied voltage, and inner diameter of the airflow nozzle. Through process optimization, this study successfully prepared core-shell nanofibers with highly regular morphologies, achieving the stable preparation of core-shell nanofibers with high uniformity. It provides core process support for their large-scale application in the semiconductor industry.

## 2. Design and Simulation Analysis of Airflow Nozzles

### 2.1. Establishment of Flow Nozzle Simulation Geometric Model

**Figure 1:**
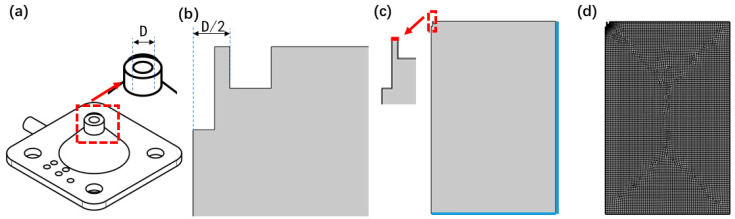
(**a**,**b**) Schematic diagram of the outer diameter of the coaxial nozzle and the inner diameter of the airflow nozzle. (**c**) Geometric model, (**d**) mesh generation.

In order to explore the influence of the inner diameter of the air nozzle on the characteristics of the airflow field and provide theoretical guidance for the air-assisted coaxial electrospinning experiment, this section conducts a simulation study based on COMSOL software. The actual geometric model of the air nozzle is shown in Figure 1a,b. Combined with the outer diameter of the coaxial needle, the inner diameter D of the air nozzle is set to five groups of variables, namely 2.70 mm, 2.90 mm, 3.14 mm, 3.32 mm, and 3.52 mm, according to the ratio of 1:1.3–1:1.7. Since the model has good axial symmetry, a two-dimensional axisymmetric model is used for the simulation to improve the computational efficiency. The simulation geometric model and the mesh generation results are shown in Figure 1c and Figure 1d respectively. The red area is set as the air inlet boundary, and the blue area is set as the air outlet boundary. The specific geometric size parameters of the model are shown in Table 1.

### 2.2. Simulation Results Analysis of the Influence of Different Inner Diameters of the Airflow Nozzle on the Airflow Field

**Figure 2:**
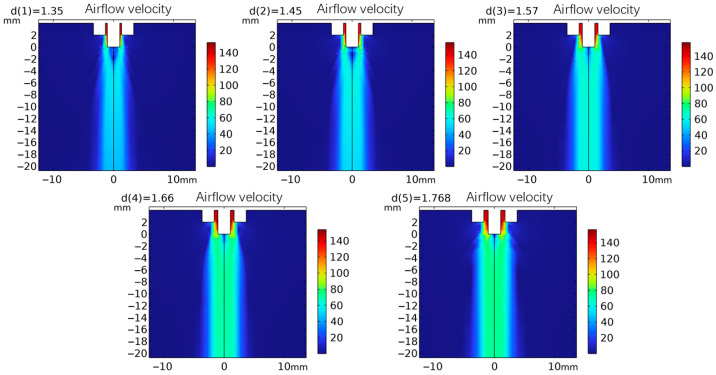
Simulation velocity nephograms of different inner diameters of airflow nozzles.

**Figure 3:**
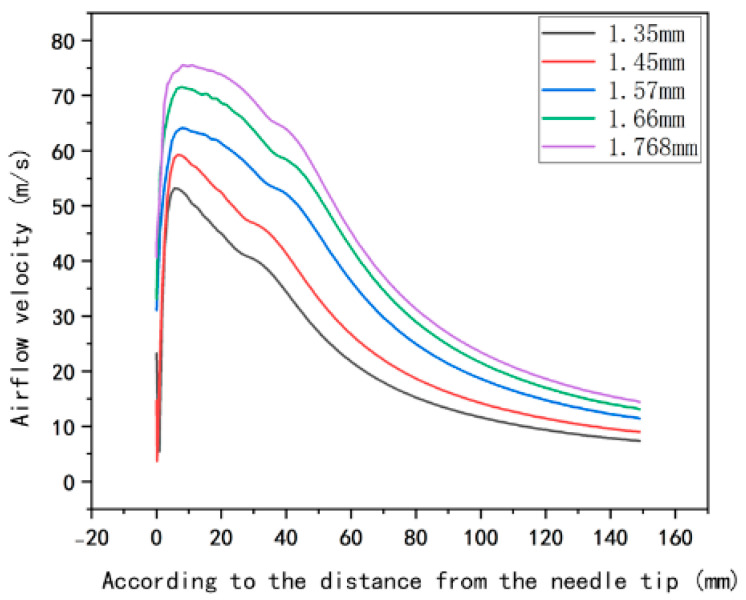
Distribution maps of the airflow velocity below the needle under different inner diameters of the airflow nozzles.

Figure 2 shows the velocity contour maps of the airflow field under different inner diameters of the airflow nozzles. It can be preliminarily seen from the figure that as the inner diameter of the airflow nozzle increases, both the peak value of the airflow velocity and the effective action range of the airflow show an increasing trend. To quantitatively characterize the influence of the inner diameter of the airflow nozzle on the axial airflow velocity distribution, this paper intercepts the velocity data in the range of 1–150 mm below the needle tip and plots the axial airflow velocity distribution curve, as shown in Figure 3.

Figure 3 shows the variation of the airflow velocity with the distance from the needle tip under different inner diameters of the airflow nozzle in airflow-assisted electrospinning. The abscissa represents the distance from the needle tip, and the ordinate represents the airflow velocity. As can be seen from the figure, the curves corresponding to each inner diameter all show a trend that the airflow velocity rapidly rises to a peak near the needle tip and then gradually decreases as the distance increases. Moreover, as the inner diameter of the airflow outlet increases, the peak value of the airflow velocity also increases accordingly. At the same distance from the needle tip, the airflow velocity with a larger inner diameter is relatively higher. This indicates that in the process of airflow-assisted electrospinning, a larger inner diameter of the airflow outlet can generate a higher airflow velocity. This has important guiding significance for electrospinning: on the one hand, a higher airflow velocity can provide a stronger stretching force, which helps to refine the fibers and improve the uniformity and quality of the fibers; on the other hand, by reasonably selecting the inner diameter of the airflow outlet, the airflow velocity can be regulated according to the required fiber characteristics, and the electrospinning process parameters can be optimized so as to effectively control the fiber structure and performance and meet the needs of different application scenarios.

## 3. Experiment

### 3.1. Experimental Materials

The polyvinyl alcohol (PVA-220, hydrolysis degree: 87~89%, Kuraray, Tokyo, Japan), polyethylene oxide (PEO-N3000, M = 400,000 g·mol^−1^, DOW, Midland, MI, USA), and deionized water were obtained from Aladdin Chemical Co. (Riverside, CA, USA). All reagents were of analytical grade and used as received.

### 3.2. Solution Preparation

At room temperature, 0.526 g of polyethylene oxide (PEO) powder was transferred into a solution vial containing 10 mL of distilled water. The mixture was then magnetically stirred continuously at room temperature for 12 h to yield a homogeneous 5 wt% PEO aqueous solution. For the preparation of PVA solution, 0.870 g of polyvinyl alcohol (PVA) was dissolved in 10 mL of distilled water at 95 °C, and heated magnetic stirring was maintained for 12 h to obtain an 8 wt% PVA aqueous solution. Multiple batches of both solutions were prepared according to experimental requirements.

### 3.3. Fabrication of Nanofiber Membrane

**Figure 4:**
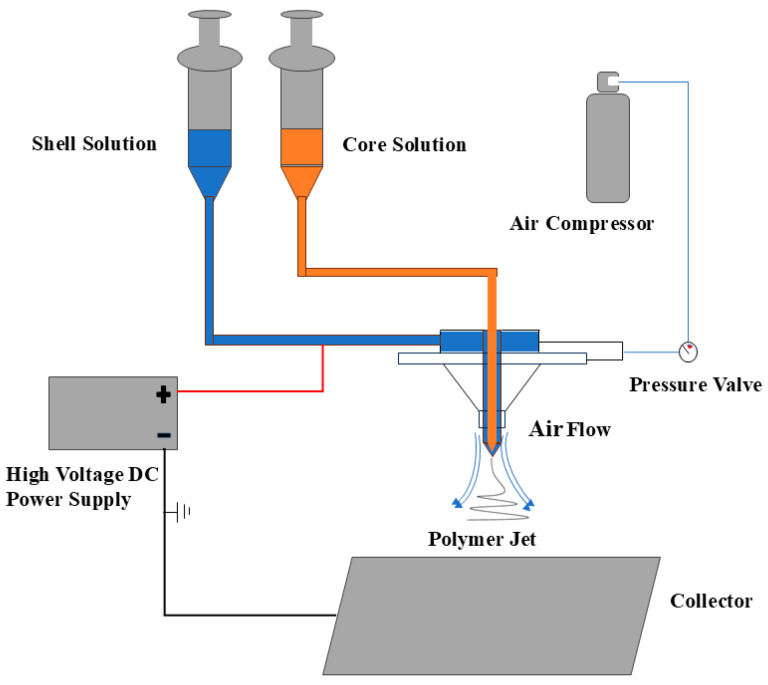
The schematic of the utilized gas-assisted coaxial electrospinning.

**Figure 5:**
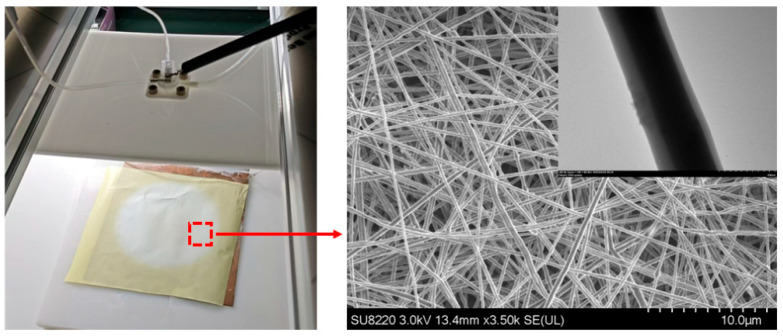
Preparation diagram of core-shell structure nanofiber membrane and TEM image of nanofibers.

The experimental platform of airflow-assisted coaxial electrospinning used in this study is shown in Figure 4, which is mainly composed of four functional modules: high-voltage power supply, compressed gas source, solution supply, and fiber collection. Under the synergistic action of the high-voltage electric field force and the airflow stretching force, the polymer solution is continuously stretched and refined to form nanofibers, which are finally deposited on the collection device, as shown in Figure 5.

All experiments in this study were conducted under constant environmental conditions: the environmental temperature was controlled at 24 °C, and the relative humidity was stabilized at 40%. To lay a foundation for the subsequent response surface experimental design, this section first carried out single-factor experiments on three factors: voltage, air pressure, and the inner diameter of the airflow nozzle. During the experiment, the process parameters were fixed as follows: the core layer used a PVA solution with a mass fraction of 8% and a liquid supply rate of 0.2 mL/h; the shell layer used a PEO solution with a mass fraction of 5% and a liquid supply rate of 1 mL/h; and the receiving distance between the needle and the collecting device was fixed at 25 cm. The remaining experimental parameters are detailed in Table 2.

### 3.4. Characterize

In this study, multi-dimensional characterization methods were employed to analyze the morphology of nanofibers and the spinning process: a field-emission scanning electron microscope (FE-SEM, model SU8220, Japan) was used to characterize the samples and obtain SEM images of the nanofibers. The diameters of more than 100 randomly selected nanofibers in the images were measured using Image J software (Version 1.53t, NIH, USA) to ensure statistical representativeness. Data analysis, such as calculating the mean and standard deviation of the diameter data, was performed, and a diameter distribution histogram was plotted using Origin Pro 2023 software (OriginLab, USA).

### 3.5. Results and Discussion

#### 3.5.1. The Effect of Working Voltage on Fiber Diameter

**Figure 6:**
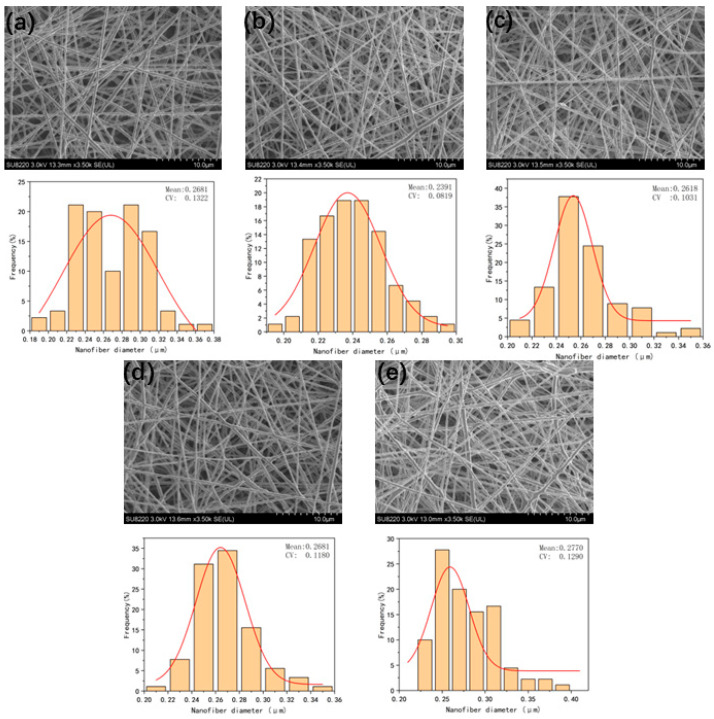
(**a**), (**b**), (**c**), (**d**), and (**e**) are the SEM images and fiber diameter distribution diagrams of fibers at voltages of 16, 17, 18, 19, and 20 kV, respectively.

**Figure 7:**
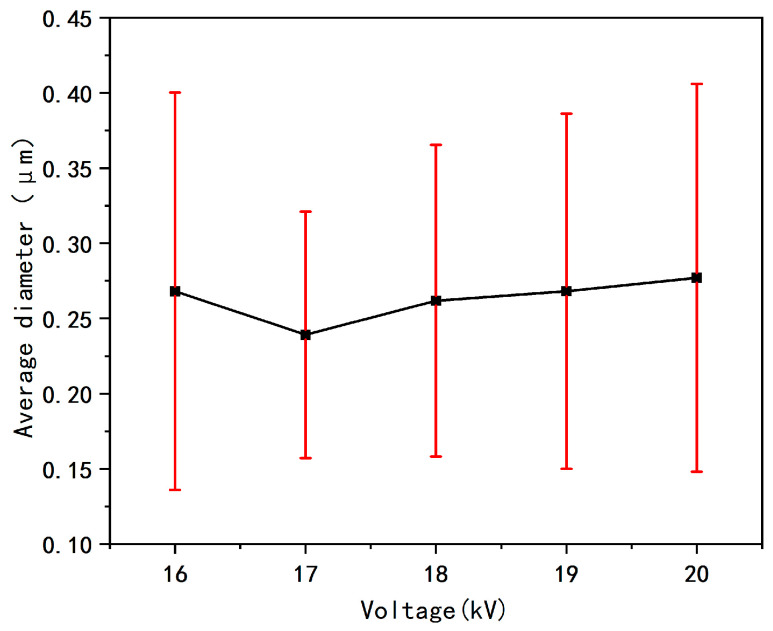
Average fiber diameter diagram at different voltages.

In Figure 6, CV represents the coefficient of variation of the fiber diameter. Figure 6 comprehensively presents the SEM morphology images of the prepared nanofiber membranes under different voltage conditions, the statistical results of the fiber diameter distribution, and the average fiber diameter data. It can be observed from Figure 7 that when the applied voltage is 16 kV, the diameter uniformity of the core-shell nanofibers is poor. The reason is that insufficient voltage leads to a weak electric field force, which is unable to fully stretch the electrospinning jet, ultimately causing jet instability.

As the applied voltage increases, the electric field strength during the electrospinning process increases synchronously, and the traction force on the jet increases, resulting in a decrease in the fiber diameter. However, when the voltage exceeds a certain threshold, the enhancement of the electric field force further increases the jet traction force and shortens the time for the fibers to reach the receiving device. As a result, the fibers are collected before being fully stretched, which instead increases the jet instability. Therefore, the average fiber diameter begins to increase, the standard deviation rises, and the uniformity of the fiber diameter distribution decreases.

In summary, within the working voltage range of 16–20 kV, the voltage selection should avoid both excessively high and low voltages to prevent the problems of an increased average fiber diameter and uneven diameter distribution. The optimal voltage under these parameter conditions is 17 kV. Under this voltage, the prepared fibers have the smallest average diameter, the lowest coefficient of variation, and the best diameter distribution uniformity.

#### 3.5.2. The Effect of Airflow Pressure on Fiber Diameter

**Figure 8:**
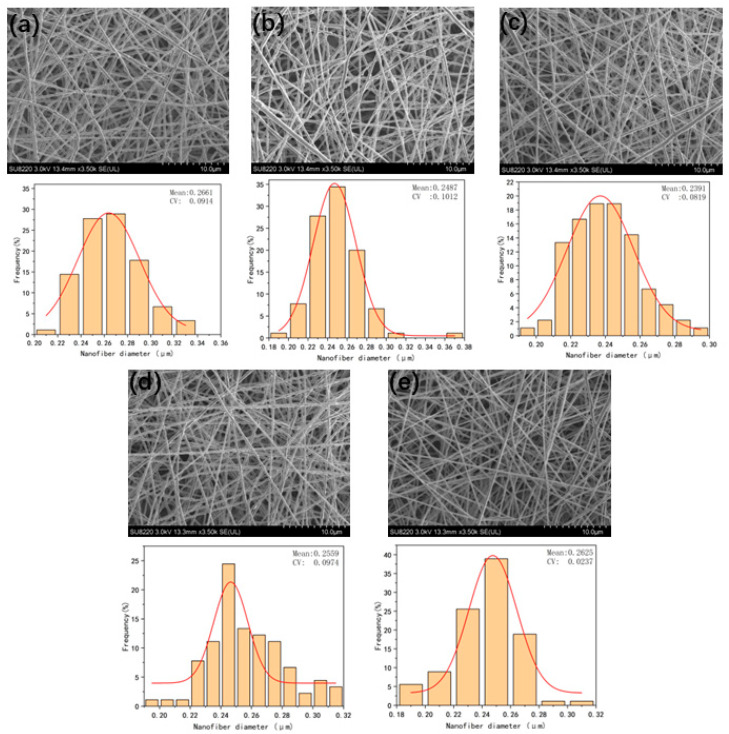
(**a**), (**b**), (**c**), (**d**), and (**e**) are the SEM images and fiber diameter distribution diagrams of the fibers under the air pressures of 5, 10, 15, 20, and 25 kPa, respectively.

**Figure 9:**
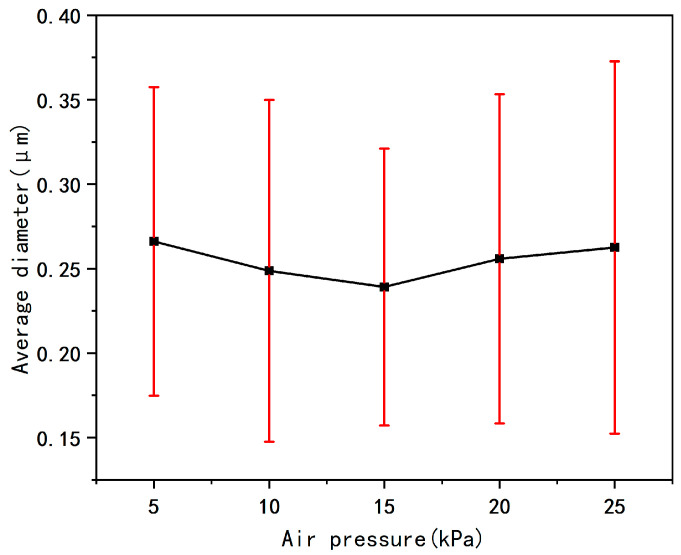
Average fiber diameter diagram at different air pressures.

Figure 8 summarizes the SEM morphology images of nanofiber membranes prepared under different air pressure conditions, the statistical results of fiber diameter distribution, and the average fiber diameter data. From the pattern in Figure 9, it can be seen that as the input air pressure increases, the traction force of the airflow on jet gradually strengthens, exerting a certain constraint on the jets, thereby enhancing the jet stability. As a result, the fiber diameter gradually decreases, and the diameter distribution becomes more uniform.

However, when the air pressure exceeds a certain threshold, the shearing effect of the airflow on the fibers significantly intensifies, and the traction force on the jets is further amplified, leading to a shorter fiber stretching time and instead increasing the jet instability. Ultimately, this is manifested as an increase in the average fiber diameter, an increase in the standard deviation, and a decrease in the uniformity of the diameter distribution.

In summary, within the air pressure range of 5 kPa to 25 kPa, parameter selection should avoid both excessively high and excessively low air pressures to prevent problems such as an increase in the average fiber diameter and non-uniform diameter distribution. The optimal air pressure under these parameter conditions is 15 kPa. Under this air pressure, both the average fiber diameter and the coefficient of variation of the prepared fibers reach the minimum values, and the uniformity of the diameter distribution is optimal.

#### 3.5.3. The Effect of the Inner Diameter of the Airflow Nozzle on Fiber Diameter

**Figure 10:**
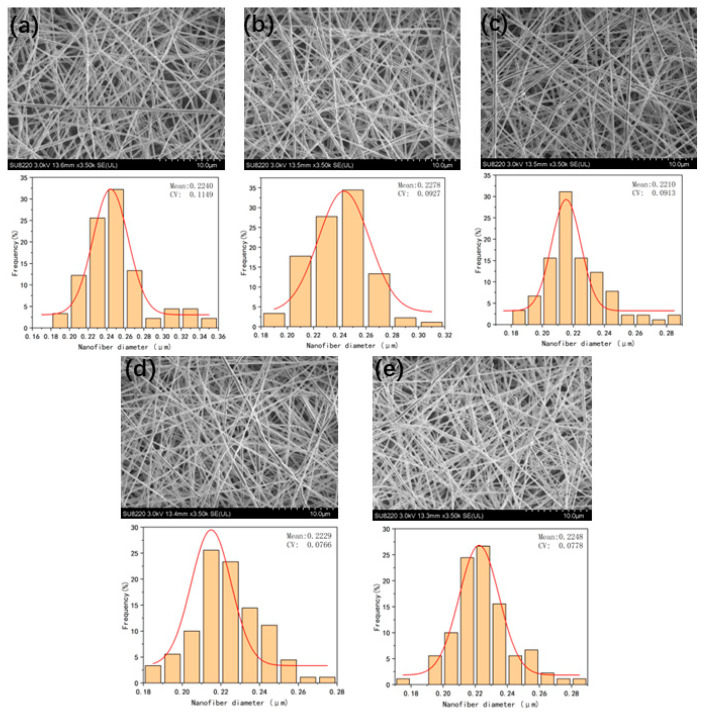
(**a**), (**b**), (**c**), (**d**), and (**e**) are the SEM images of fibers and the fiber diameter distribution maps under the inner diameters of air nozzles of 2.7, 2.9, 3.12, 3.32, and 3.52 mm respectively.

**Figure 11:**
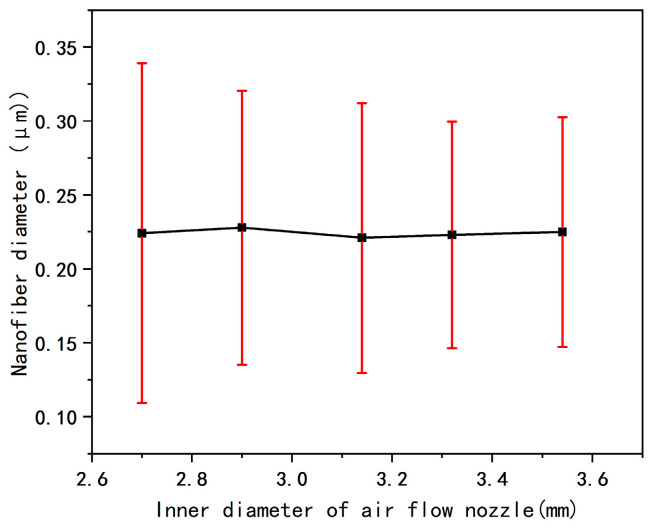
Average fiber diameter diagram at different inner diameters of the airflow nozzle.

Figure 10 shows the SEM morphology, fiber diameter distribution statistical results, and average fiber diameter data of the nanofiber membranes prepared under different inner diameters of the airflow nozzles. According to the pattern presented in Figure 11, it can be concluded that as the inner diameter of the airflow nozzle increases, the air intake volume per unit time increases accordingly, which further enhances the airflow velocity at the nozzle outlet, strengthens the traction force on the jet, effectively suppresses the interference of ambient airflow disturbances on the jets, improves the stability of the initial jets, and finally results in finer and denser fibers.

However, when the inner diameter of the airflow nozzle increases to a certain threshold, the velocity and pressure at the airflow outlet further increase, and the traction force on the jets is excessively amplified, which instead increases the instability of the jets. Finally, it is manifested as an increase in the average fiber diameter, an increase in the standard deviation, and a decrease in the uniformity of the diameter distribution.

In summary, within the range of the nozzle inner diameter from 2.7 mm to 3.52 mm, the parameter selection needs to avoid both excessively large and small values to prevent the problems of an increase in the average fiber diameter and uneven diameter distribution. The optimal inner diameter of the nozzle under these parameter conditions is 3.32 mm. Under this inner diameter, both the average fiber diameter and the coefficient of variation of the prepared fibers reach the minimum values, and the uniformity of the diameter distribution is the best.

## 4. Response Surface Analysis and Experimental Verification

### 4.1. Experimental Design of Response Surfaces

A three-factor and three-level experimental design was employed, and the specific parameter settings are shown in Table 3. This study was based on the Box-Behnken design (BBD) method, and a total of 17 groups of coded combination optimization experiments of process parameters were designed. After conducting the experiments according to the designed combinations of process parameters for each group, the measurement method established in the single-factor experiment was used to measure the coefficient of variation of the fiber diameter under the corresponding conditions. The obtained experimental results were organized and filled into the corresponding cells of the design matrix, and finally, the experimental design table was obtained, as shown in Table 4.

### 4.2. Analysis of Response Surface Results

The regression model can optimize process parameters and predict response indicators, ultimately obtaining the best regression fitting equation. Using Design-Expert software (Design-Expert 11.0), a response surface regression model and a mathematical model of response were indicators constructed for the average fiber diameter data obtained from the experiment, as shown in Table 5. The sum of squares represents the amount of variation explained by the model, reflecting the strength of the relationship between factors and response variables. In the analysis of variance, these sums of squares are used for calculation. Then, the mean square values are used for significance testing. The F-value of this model is 7.99, indicating that the model is significant. By comparing the *p*-value of the above model with the significance level, the *p*-value is used to analyze the significance of the studied model. A *p*-value greater than 0.1 indicates the response model is not significant. When the *p*-value is less than 0.05, it means that the model is at a significant level. When the *p*-value is less than 0.0001, it means that the response model reaches a highly significant level. The analysis results show that the interaction between air pressure and the inner diameter of the airflow nozzle (AC), the square of air pressure (A^2^), and the square of the inner diameter of the airflow nozzle (C^2^) are important model factors, indicating that they have a significant impact on the uniformity of fiber diameter. By comparing the mean square values, it is found that the interaction intensity of process parameters on the uniformity of nanofiber diameter is AC > BC > AB. In this experiment, the mathematical model of response indicators established by the response surface method is:

$$\begin{array}{l} Y=3.68096-0.0281205\times A-0.09192\times B-1.56413\times C\\ +0.00012\times A\times B+0.005875\times A\times C+0.0055\times B\times C\\ +0.0002031\times A^{2}+0.0021525\times B^{2}+0.206937\times C^{2} \end{array}$$

**Figure 12:**
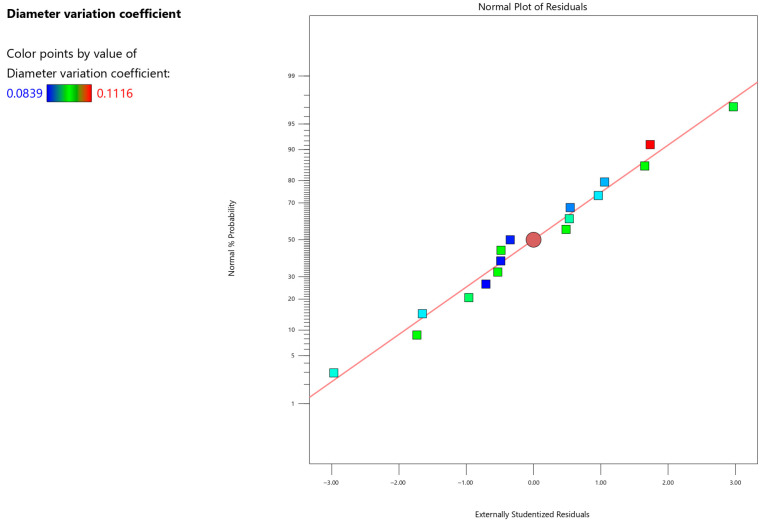
The residual of the average nanofiber diameter.

*Y* is the coefficient of variation of the diameter of the core-shell nanofiber membrane. The remaining normal probability plot is used to verify the reliability of the model by observing the data to determine if the data basically follows a linear distribution. The normal probability graph is shown in Figure 12. The *X*-axis of Figure 12 represents the theoretical quantiles of the standard normal distribution. The *Y*-axis represents the cumulative probability of data points under a standard normal distribution. The data in Figure 12 is finally displayed as a straight line, which indicates that the accuracy of the model is reliable.

**Figure 13:**
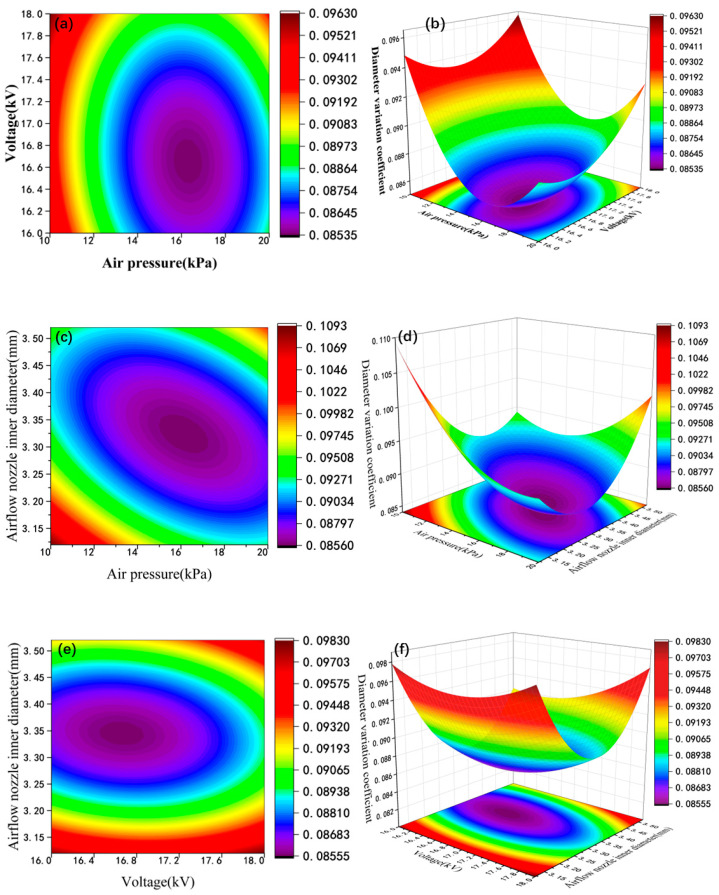
(**a**) Contour plots of air pressure and voltage; (**b**) response surface plots of air pressure and voltage; (**c**) contour plots of air pressure and inner diameter of airflow nozzle; (**d**) response surface plots of air pressure and inner diameter of airflow nozzle; (**e**) contour plots of voltage and inner diameter of airflow nozzle; (**f**) response surface plots of voltage and inner diameter of airflow nozzle.

The influence of different process parameters on the coefficient of variation of the diameter of nanofibers was studied through response surface analysis. Then, the response surface curves were drawn using Design-Expert software, as shown in Figure 11. The process parameters include air pressure, working voltage, and the inner diameters of the airflow nozzles. Figure 13a,b show the two-dimensional and three-dimensional response surface profiles of the interaction between air pressure and voltage. As the air pressure increases, the coefficient of variation of the diameter of core-shell nanofibers decreases relatively. This is because, as the input air pressure and voltage increase, the traction force of multiple jets increases, making the jets subject to a certain binding force, and the jets become more stable. As a result, the fiber diameter becomes smaller, and the diameter distribution becomes more uniform. However, higher air pressure and voltage will also intensify the interference of the airflow field, affecting the stable formation of the Taylor cone and the flow trajectory of the jets. The above-mentioned phenomena are not conducive to the uniformity of the fiber diameter distribution. Therefore, it can be seen that air pressure has a significant impact on the diameter uniformity of nanofibers. Figure 13c,d show the two-dimensional and three-dimensional response surface profiles of the interaction between air pressure and the inner diameter of the air nozzle. From these profiles, it can be seen that both the air pressure and the inner diameter of the air nozzle have a significant impact on the diameter uniformity of core-shell structured nanofibers. When the inner diameter of the air nozzle and the air pressure are relatively small, the air flow velocity and range under the coaxial nozzle are relatively small, and the airflow cannot provide sufficient binding force to stabilize the jets. As the air pressure and the inner diameter of the air nozzle increase, the binding force of the airflow the jets also increases. At the same time, the airflow range is wider, reducing the interference of external airflow, increasing the stability of the jets, making the fiber diameter more uniform, and reducing the coefficient of variation of the diameter. As the air pressure further increases, the instability of the airflow field increases. Meanwhile, due to the excessive pulling force on the jets, the jets break, the diameter uniformity of the fibers deteriorates, and the coefficient of variation of the diameter increases. Figure 13e,f show the two-dimensional and three-dimensional response surface profiles of the interaction between the working voltage and the inner diameter of the air nozzle. When the inner diameter of the air nozzle and the working voltage are relatively small, due to the insufficient electric field force and the small airflow range, it is easy to be interfered with by external airflow. The diameter uniformity of the fibers is relatively poor, and the coefficient of variation of the diameter is relatively large. As the working voltage and the inner diameter of the air nozzle increase, the electric field force provides a large pulling force for the jets. At the same time, the airflow area becomes larger, reducing interference of external airflow. The jets are relatively stable, the diameter uniformity of the fibers is better, and the coefficient of variation of the diameter is smaller. However, when the voltage is too large, the electric field force is too large, and the fibers may break before being collected by the collection device. The diameter uniformity of the fibers deteriorates, and the coefficient of variation of the diameter increases.

### 4.3. Optimization and Validation

The process parameter equations in the RSM were analyzed using Design-Expert software. The extreme points of the response indicators were automatically explored through optimization to determine the optimal process parameter configuration. The configuration parameters were a gas pressure of 10 kPa, a working voltage of 16.71 kV, and an inner diameter of the airflow nozzle of 3.42 mm. When these were used as the optimal parameter configuration, the coefficient of variation of the nanofiber diameter was 0.0920. Repeated operations were carried out using this parameter configuration. The optimal results were verified through experiments, and the coefficient of variation of the diameter obtained in the repeated experiments was 0.0941. Compared with the optimal result of 0.092, the deviation was only about 2.23%. This indicates that the regression equation constructed by RSM is relatively accurate and can be used for process optimization in the air-assisted coaxial electrospinning experiment.

## 5. Conclusions

This study addresses the problems of traditional coaxial electrospinning technology in preparing core-shell nanofibers, such as unstable electric fields, poor diameter uniformity, and limited adaptability to functional materials. The GACES method is proposed, and the systematic optimization of process parameters is achieved by combining COMSOL finite element simulation and RSM.

The influence of the inner diameter of the gas nozzle on the coaxial pinhole on the gas flow field distribution is analyzed through COMSOL simulation, and the internal mechanism of improving fiber uniformity by the synergistic electrostatic stretching of the gas flow is revealed. Further, the multi-parameter optimization of gas pressure, working voltage, and gas nozzle inner diameter is carried out using RSM, and the optimal process combination is determined as follows: gas pressure of 10 kPa, voltage of 16.71 kV, and gas nozzle inner diameter of 3.42 mm. Under these conditions, the coefficient of variation of the diameter of the core-shell nanofibers is as low as 9.2%, and the morphology is highly regular, which verifies the accuracy of the RSM regression equation (the deviation between the experimental value and the predicted value is only 2.23%). This study not only breaks through the technical bottlenecks of traditional electrospinning and realizes the stable preparation of highly uniform core-shell nanofibers but also provides key process support for its large-scale application in fields such as semiconductor packaging and flexible circuits. In the future, the application of GACES technology in high-viscosity/low-conductivity precursor systems can be further expanded to promote the development of flexible electronics and photoelectric detection technology and lay the foundation for the research and development of next-generation semiconductor devices.

In future research, we will focus on regulating the diameter of the core layer of core-shell structured nanofibers through gas-assisted coaxial electrospinning technology, as well as achieving synchronous and precise control of the diameters of the core-shell and the shell layer. To achieve this goal, further theoretical analysis and numerous experiments are still required.

## Figures and Tables

**Table 1:** Core dimensions of the model.

Coaxial Nozzle Outer Diameter	Working Pressure	Airflow Environment Zone
2.08 mm	15 kPa	Pi × (100 mm)^2^ × 150 mm

**Table 2:** Process parameters of a single-factor experiment.

		Voltage (kV)	Air Pressure (kPa)	Inner Diameter of Airflow Nozzle (mm)
	
Single-factor experiment of voltage		16/17/18/19/20	15	3.12
Single-corfactor experiment of air pressure		17	5/10/15/20/25	3.12
Single-factor experiment on the inner diameter of the airflow nozzle		17	15	2.7/2.9/3.12/3.32/3.52

**Table 3:** Coding levels of electrospinning process parameters.

Factor	Code		Level	
		−1	0	1
Air pressure (kPa)	A	10	15	20
Voltage (kV)	B	16	17	18
Inner diameter of the airflow nozzle (mm)	C	3.12	3.32	3.52

**Table 4:** Experimental schemes and results.

Number	Air Pressure (kPa)	Voltage (kV)	Inner Diameter of the Airflow Nozzle (mm)	CV of Diameter
1	10	16	3.32	0.0917
2	20	16	3.32	0.0903
3	10	18	3.32	0.0948
4	20	18	3.32	0.0958
5	10	17	3.12	0.1116
6	20	17	3.12	0.0904
7	10	17	3.52	0.0964
8	20	17	3.52	0.0987
9	15	16	3.12	0.0986
10	15	18	3.12	0.0974
11	15	16	3.52	0.0931
12	15	18	3.52	0.0963
13	15	17	3.32	0.0875
14	15	17	3.32	0.0839
15	15	17	3.32	0.0849
16	15	17	3.32	0.0888
17	15	17	3.32	0.0845

**Table 5:** Analysis of variance and significance check of regression equation for nanofiber diameter uniformity.

Source	Sum of Squares	Mean Square	F-Value	*p*-Value	
Model	0.0007	0.0001	7.99	0.0060	significant
A	0.0000	0.0000	4.93	0.0619	
B	0.0000	0.0000	1.49	0.2623	
C	0.0000	0.0000	2.41	0.1645	
AB	1.440 × 10^−6^	1.440 × 10^−6^	0.1523	0.7079	
AC	0.0001	0.0001	14.61	0.0065	significant
BC	4.840 × 10^−6^	4.840 × 10^−6^	0.5120	0.4974	
A^2^	0.0001	0.0001	11.48	0.0116	significant
B^2^	0.0000	0.0000	2.06	0.1940	
C^2^	0.0003	0.0003	30.52	0.0009	significant

## Data Availability

The data that support the findings of this research are available from the corresponding author upon reasonable request.

## References

[1] Shen Z., Wu H., Liu C., Liu Z., Jiang Y., Wang T., Zhou P. (2026). Wafer-scale Monolayer Dielectric Integration on Atomically Thin Semiconductors. Nat. Mater..

[2] Huang T., Cao J., Yu Z., Zhang Y., Xu W., Li X., Peng C., Sun W., Yang G., Wu W. (2026). Demonstration of InSnO Thin-Film Transistors with Superior Uniformity and Reliability Utilizing SiO_2_ Passivation. J. Semicond..

[3] Okamoto N., Wang X., Morita K., Kato Y., Alom M.M., Magari Y., Furuta M. (2024). Uniformity and Reliability of Enhancement-Mode Polycrystalline Indium Oxide Thin Film Transistors Formed by Solid-Phase Crystallization. IEEE Electron Device Lett..

[4] Zhou J., Zhang Q., Gong J., Lu Y., Liu Y., Abbasi H., Qiu H., Kim J., Lin W., Kim D. (2024). Wafer-Scale Semiconductor Grafting: Enabling High-Performance, Lattice-Mismatched Heterojunctions. arXiv.

[5] Liu J., Yu Y., Liu J., Li T., Li C., Zhang J., Hu W., Liu Y., Jiang L. (2022). Capillary Confinement Crystallization for Monolayer Molecular Crystal Arrays. Adv. Mater..

[6] Lo S., Lin Y. (2005). The prediction of wafer surface non-uniformity using FEM and ANFIS in the chemical mechanical polishing process. J. Mater. Process. Technol..

[7] Chen Z.Z., Yuan H., Wang H.G., Zhu Y., Chen X.L., Zhang Z.R., Zhang W.J., Zhang Y.M. (2024). Defect Density Control in 4H-SiC Epilayers for 10 kV Power Devices. Mater. Sci. Semicond. Process..

[8] Wu Y., Du H., Zhang Z., Cong L., Xu S., Sun S. (2022). Fabrication of In_2_O_3_/SnO_2_-Coaxial-Electrospinning Fiber and Investigation on Its Formaldehyde Sensing Properties. Acta Mater. Compos. Sin..

[9] Kimura T., Hachiya K., Sagawa T. (2023). Ligand-Free CsPbBr_3_@TiO_2_ Core-Shell Nanofibers with Enhanced Carrier Transport. J. Electrochem. Soc..

[10] Yang G., Yan W., Zhang Q., Shen S., Ding S. (2013). One-dimensional CdS/ZnO Core/Shell Nanofibers via Single-Spinneret Electrospinning. Nanoscale.

[11] Kim M., Jo S.B., Park J.H., Cho K. (2015). Flexible Lateral Organic Solar Cells with Core–Shell Structured Organic Nanofibers. Nano Energy.

[12] Han D., Yang L., Hu Z., Du Z., Wang Y., Yuan Z., Wang Q., Artemyev M., Tang J. (2020). Sharp Fluorescence Nanofiber Network of CdSe/CdS Core-Shell Nanoplatelets. ScienceAsia.

[13] Kim J.R., Choi S.W., Jo S.M. (2005). Electrospun PVDF-based fibrous polymer electrolytes for lithium ion polymer batteries. Electrochim. Acta.

[14] Zhang S., Dong X., Xu S., Wang J. (2007). TiO_2_@SiO_2_ Sub-Micro Coaxial Cables via Static Electricity Spinning. Acta Chim. Sin..

[15] Li D., Wang Y., Xia Y. (2003). Electrospinning of Polymeric and Ceramic Nanofibers as Uniaxially Aligned Arrays. Nano Lett..

[16] Sun Z., Zussman E., Yarin A.L., Wendorff J.H., Greiner A. (2003). Compound Core–Shell Polymer Nanofibers by Co-Electrospinning. Adv. Mater..

[17] Li D., Xia Y. (2004). Direct Fabrication of Composite and Ceramic Hollow Nanofibers by Electrospinning. Nano Lett..

[18] Loscertales I.G., Barrero A., Guerrero I., Cortijo R., Marquez M., Gañán-Calvo A.M. (2002). Micro/Nano Encapsulation via Electrified Coaxial Liquid Jets. Science.

[19] Ryu H.I., Koo M.S., Kim S., Kim S., Park Y.A., Park S.M. (2020). Uniform-thickness electrospinning nanofibers mat production system based on real-time thickness measurement. Sci. Rep..

[20] Shao Z., Wang Q., Gui Z., Shen R., Chen R., Liu Y., Zheng G. (2024). Electrospun bimodal nanofibrous membranes for high performance, multifunctional, and light-weight air filtration, A review. Sep. Purif. Technol..

[21] Hwang S.H., Song J.Y., Ryu H.I., Oh J.H., Lee S., Lee D., Park D.Y., Park S.M. (2023). Correction to: Adaptive Electrospinning System Based on Reinforcement Learning for Uniform-Thickness Nanofiber Air Filters. Adv. Fiber Mater..

[22] Zhang R., Chen X., Wang H., Zeng J., Zhang X., Chen X. (2024). Study on uniformity of multi-needle electrostatic spinning by auxiliary flow field. Micro Nano Lett..

[23] Zhang R., Chen X., Wang H., Sun J., Huang S., Zhang X., Long J. (2025). Study on Deposition of Coaxial Electrospinning Fibers by Coaxial Auxiliary Flow Field. Polymers.

[24] Abdolbaghian H., Bazgir S. (2022). Fabrication and characterization of gas-assisted core-shell hydrogel nanofibers as a drug release system with antibacterial activity. Eur. Polym. J..

